# Hot Electrons, Hot Holes, or Both? Tandem Synthesis of Imines Driven by the Plasmonic Excitation in Au/CeO_2_ Nanorods

**DOI:** 10.3390/nano10081530

**Published:** 2020-08-04

**Authors:** Ivo F. Teixeira, Mauricio S. Homsi, Rafael S. Geonmonond, Guilherme F. S. R. Rocha, Yung-Kang Peng, Ingrid F. Silva, Jhon Quiroz, Pedro H. C. Camargo

**Affiliations:** 1 Department of Chemistry, Federal University of São Carlos, São Carlos 13565-905, Brazil; 2 Department of Fundamental Chemistry, Institute of Chemistry, University of São Paulo, São Paulo 05508-000, Brazil; mauriciosamuel.homsi@usp.br (M.S.H.); rafaelsg31@gmail.com (R.S.G.); guilhermefrrocha@gmail.com (G.F.S.R.R.); 3 Department of Chemistry, City University of Hong Kong, Yeung Kin Man Academic Building, Hong Kong, China; ykpeng@cityu.edu.hk; 4 Department of Chemistry, ICEx, Universidade Federal de Minas Gerais, Belo Horizonte 31270-901, Brazil; ingridfs@ufmg.br; 5 Department of Chemistry, University of Helsinki, A.I. Virtasen aukio 1, 00100 Helsinki, Finland; jhon.quiroz@helsinki.fi

**Keywords:** tandem, oxidative coupling, Au NPs, CeO_2_ nanorods, localized surface plasmon resonance, nanocatalysis

## Abstract

Solar-to-chemical conversion via photocatalysis is of paramount importance for a sustainable future. Thus, investigating the synergistic effects promoted by light in photocatalytic reactions is crucial. The tandem oxidative coupling of alcohols and amines is an attractive route to synthesize imines. Here, we unravel the performance and underlying reaction pathway in the visible-light-driven tandem oxidative coupling of benzyl alcohol and aniline employing Au/CeO_2_ nanorods as catalysts. We propose an alternative reaction pathway for this transformation that leads to improved efficiencies relative to individual CeO_2_ nanorods, in which the localized surface plasmon resonance (LSPR) excitation in Au nanoparticles (NPs) plays an important role. Our data suggests a synergism between the hot electrons and holes generated from the LSPR excitation in Au NPs. While the oxygen vacancies in CeO_2_ nanorods trap the hot electrons and facilitate their transfer to adsorbed O_2_ at surface vacancy sites, the hot holes in the Au NPs facilitate the α-H abstraction from the adsorbed benzyl alcohol, evolving into benzaldehyde, which then couples with aniline in the next step to yield the corresponding imine. Finally, cerium-coordinated superoxide species abstract hydrogen from the Au surface, regenerating the catalyst surface.

## 1. Introduction

Photocatalysis can initiate or accelerate chemical reactions by the light–matter interaction, which can simultaneously solve the problems about solar energy conversion and storage [1]. Photocatalysts can directly convert solar energy into chemical energy and simultaneously accomplish solar energy conversion and storage objectives [1]. Metal/semiconductor hybrid photocatalysts have emerged as one of the most promising catalytic systems to promote solar-to-chemical conversion. Therefore investigation of the synergistic effects promoted by light in these hybrid photocatalytic systems is of paramount importance for the design of new photocatalysts containing plasmonic components that can enable improved performance and selectivity via reaction pathways that are not accessible in semiconducting nanoparticles (NPs) or external heating.

The tandem conversion of alcohols into imines, via alcohol oxidation followed by an amine coupling, is of great interest in organic and green chemistry as it can be carried out under mild conditions, alcohols are widely available, and only hydrogen or water are generated as byproducts [2,3,4,5,6]. CeO_2_ NPs have been effective as catalysts towards this tandem transformation, where a correlation between oxygen vacancies (as enabled by distinct nanoparticle shapes) and the catalytic activity has been established [6,7,8]. For instance, CeO_2_ nanorods have shown improved performance relative to nanocubes and nanoctahedra due to the exposure of (110) surface facets that enable a greater concentration of oxygen vacancies [7,8].

It has been shown that the nonradiative decay following localized surface plasmon resonance (LSPR) excitation in plasmonic metals leads to hot electrons and holes, that can participate in the electronic or vibrational activation of species close to the surface [9,10]. This provides sufficient energy to initiate, accelerate, and/or control molecular transformations [9,10]. For metals such as Ag, Au, Al, and Cu, the LSPR excitation can take place in the visible range [11,12]. Interestingly, the combination of CeO_2_ and plasmonic NPs, such as Au, can provide opportunities for not only achieving improved performance but also opening up reaction pathways under light-excitation that are not accessible via external heating [13,14,15,16,17,18].

In this work, we unravel the performance and underlying reaction pathway in the visible-light-driven tandem oxidative coupling of benzyl alcohol and aniline employing Au/CeO_2_ nanorods as catalysts. We propose an alternative reaction pathway for this transformation that leads to improved efficiencies relative to individual CeO_2_ nanorods, in which the LSPR excitation in Au NPs played an important role. Our data suggests a synergism between the hot electrons and holes generated from the LSPR excitation in Au NPs, which acted in combination with the oxygen vacancies in the CeO_2_ to improve catalytic activities.

## 2. Experimental Section

### 2.1. Au Nanoparticles Synthesis

Spherical gold nanoparticles with 10–20 nm diameter were synthesized using HAuCl_4_, deionized water, ascorbic acid and polyvinylpyrrolidone (PVP). To a 50 mL round bottom flask were added 6 mL deionized water, 35 mg PVP and 60 mg ascorbic acid, all the solids were dissolved and then the mixture was heated to 90 °C. After 10 min at 90 °C, 1 mL of 3 mmol/L HAuCl_4_ solution (5.1 mg in 5 mL of water) was added as fast as possible under intense stirring. After that, the reaction took 3 h at 90 °C and stirring. The obtained Au nanoparticles were washed with 100 mL of water and then suspended in ethanol [19].

### 2.2. Au/CeO_2_ Photocatalyst Synthesis 

CeO_2_ nanorods were synthesized with Ce(NO_3_)_2_·6H_2_O, sodium hydroxide and deionized water. A concentrated NaOH solution was prepared by adding 56 g NaOH into 100 mL deionized water, the solution was cooled to ambient temperature before continuing. After that, 19.9 g Ce(NO_3_)_2_·6H_2_O was added to the NaOH solution and stirred for 30 min, the resultant mixture was transferred into a 150 mL Teflon-lined autoclave and sealed. In order to produce nanorods, the autoclave was kept at 110 °C for 24 h. After that the solution obtained was washed with 1 L deionized water and 100 mL acetone and then transferred to a porcelain crucible, dried at 110 °C for 6 h and calcined for 4 h at 400 °C [20]. Controlled morphology CeO_2_ was impregnated with Au nanoparticles using the incipient wetness method. After impregnation, the catalyst was dried at 90 °C overnight and stored.

### 2.3. Catalyst Characterization

Transmission electron microscopy (TEM) images were taken on a JEOL 2100 electron microscope (JEOL, Tokyo, Japan) operating at an accelerating voltage of 200 kV. The samples were prepared to TEM observations by dispersion in ethanol using an ultrasound bath and deposited onto holey-carbon copper grids. The solid-state UV−vis spectra were recorded on a Shimadzu UV-2600 PC spectrophotometer (Shimadzu Corporation, Kyoto, Japan). Wide-angle powder X-Ray Diffraction (XRD) data were recorded in a Rigaku Miniflex diffractometer (Rigaku Corporation, Tokyo, Japan) with Cu Kα radiation (λ = 1.54 Å). The data of 2*θ* from 10 to 90° were collected with a step size of 0.02° and acquisition time of 1 s. The diffraction patterns have been indexed by comparison with the Joint Committee on Powder Diffraction Standards (JCPDS) files. The concentrations of Au were determined by atomic absorption spectrometry (AA–Hitachi-Z8200 spectrometer, Hitachi, Japan). The surface area values were obtained by the BET method and the BJH model was used to determine the pore size distributions. The N_2_ adsorption and desorption measurements were carried out at 77 K using an Autosorb iQ2 Quantachrome Autosorb Equipment (Quantachrome, Boynton Beach, FL, USA).

### 2.4. Photocatalytic Tests

All the photocatalytic tests were conducted in a 100 mL pressurized photoreactor. In a typical photocatalytic experiment 2.5 mL mesitylene, 80 μL benzyl alcohol, 100 μL aniline, 50 mg catalyst are mixed. With the reactor sealed to prevent solvent evaporation, the catalyst was suspended in an ultrasonic bath and then pressurized with 1 bar O_2_. Except when indicated, all the tests took 48 h at 50 °C with visible spectrum light (300 W) incidence under magnetic stirring. This temperature was chosen based on the best yields reported in the literature for this reaction [21]. The reaction products were analyzed by gas chromatography (GC). Two aliquots (500 μL) were collected for each reactor batch, the first one before the O_2_ loading (*t* = 0) and the second one at the end of the reaction, typically 48h. In both cases, the catalyst was removed by centrifugation (11,000 rpm, 1 min). Both samples were injected in a Shimadzu GC-2010 Plus gas chromatograph. The GC is equipped with a flame ionization detector, which enabled the separation and quantification of all reaction products using an RTX-Wax (30 m × 0.25 mm × 0.25 μm) chromatographic column. The following temperature program was used during the analysis: 50 °C for 3 min; 15 °C min^−1^ until 220 °C.

### 2.5. ^31^P NMR Measurement of TMP-Adsorbed Samples

A sample of 200 mg of CeO_2_ was placed in a glass tube and removed surface adsorbed water at 200 °C for 2 h under vacuum. After cooling down to room temperature, around 300 μmol/catalyst g of trimethylphosphine (TMP) was then introduced. It was allowed around 15 min to reach the steady state for adsorption between TMP and catalyst surface. The sample was then vacuumed at room temperature for 5 min and thus excess non-adsorbed TMP molecules were removed gently without affecting all the chemisorbed peaks. The sample tube was then flame sealed for storage and transferred to Bruker 4mm ZrO_2_ rotor with a Kel-F endcap in a glove box under nitrogen atmosphere before NMR characterization. The solid state magic angle spinning (MAS) NMR experiments were carried out using a Bruker Avance III 400WB spectrometer at room temperature. The radiofrequency for decoupling was 59 kHz. The spectral width was 400 ppm, from 200 to −200 ppm. The number of scannings was 800. The ^31^P chemical shifts were reported relative to 85% aqueous solution of H_3_PO_4_, with NH_4_H_2_PO_4_ as a secondary standard (0.81 ppm). 

### 2.6. XPS Analysis

Photoemission studies were conducted in a SPECSLAB II instrument equipped with a Ultra-high vacuum (UHV) chamber where the base pressure was less than 5 × 10^−9^ Torr. The instrument has a hemispherical electron energy analyzer PHOIBOS-Has 3500 150 with a 9-channels detector operating at 12 kV, pass energy (Epass) = 40 eV, 0.2 eV energy step and an Al Kα X-ray source. The samples were placed on stainless steel sample-holders and were placed at the XPS prechamber and held there for 2 h in a vacuum. The collected spectra were adjusted with the CasaXPS 2.3.13 software. The C 1s peak was used as an internal standard for the charge correction (284.5 eV). A Shirley-type background was used [22]. The analysis of the Ce 3d spectra was performed by adjusting 10 components, where 4 components correspond to the Ce^3+^ oxidation state (u0, v0, u’, v’) and the 6 components correspond to the Ce^4+^ oxidation state (u, v, u”, v”, u”’, v”’) [23]. The intensity ratio of Ce 3d_5_*_/_*_2_/Ce 3d_3_*_/_*_2_ doublets was set to 1.5 and the energy position was allowed to vary ~2eV around the expected values [24], avoiding the inversion of positions.

## 3. Results and Discussion

**Figure 1:**
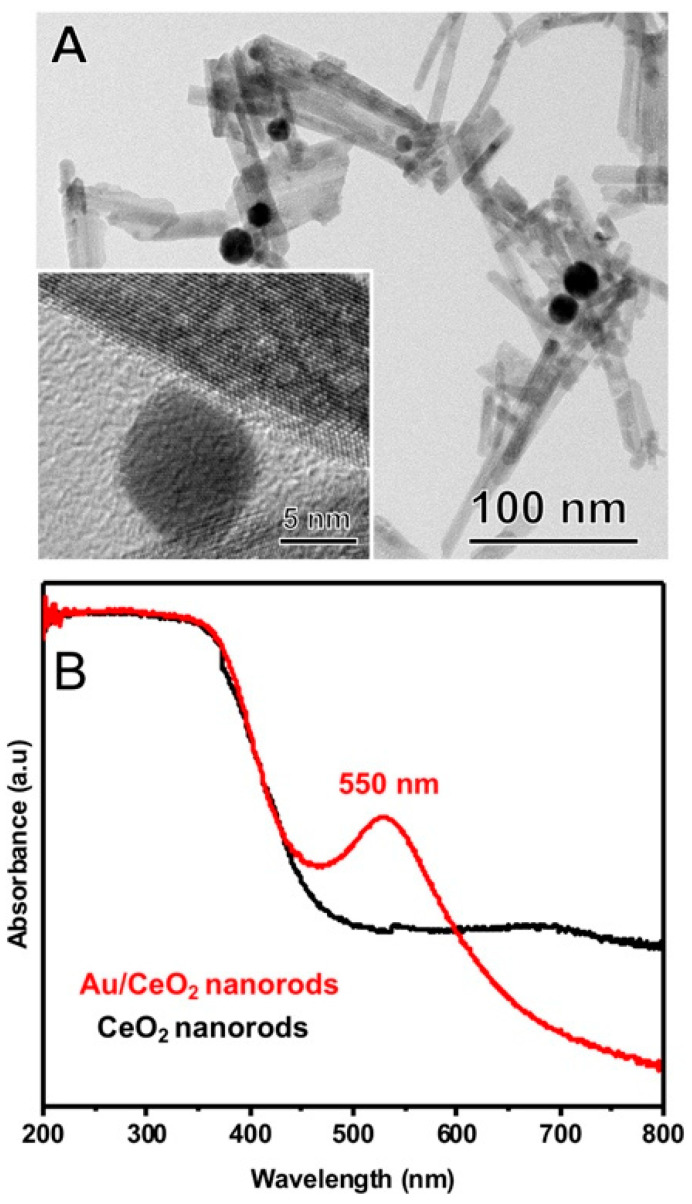
(**A**) HRTEM images for the Au/CeO_2_ nanorods. (**B**) UV−vis extinction spectra for CeO_2_ and Au/CeO_2_ nanorods (black and red traces, respectively).

The synthesis of Au NPs supported on the CeO_2_ nanorods (Au/CeO_2_) was performed by wet impregnation [20]. Figure 1A shows high-resolution transmission electron microscopy (HRTEM) images of the Au/CeO_2_ nanorods. The Au NPs presented sizes in the 10–20 nm range. The CeO_2_ nanorods were around 10 and 200 nm in width and length, respectively (Figure S1). The Au loading as determined by atomic absorption spectroscopy corresponded to 0.64 wt.%. X-ray diffractometry (Figure S2) showed only peaks assigned to the CeO_2_ phase. The UV−vis spectra of the CeO_2_ and Au/CeO_2_ samples are depicted in Figure 1B (black and red traces, respectively). It can be observed that CeO_2_ samples absorb light in the near-visible UV (<450 nm) [8]. Upon Au deposition, a band in the visible (≈550 nm) appears due to the LSPR dipolar mode in Au NPs [8,12].

Raman and XPS spectroscopies were employed to probe the metal-support interactions and oxygen vacancy properties in CeO_2_ and Au/CeO_2_ nanomaterials. It is well-known that oxygen vacancies are structural defects that can adsorb O_2_ molecules and are correlated to oxygen mobility and storage capacity [25]. The Raman spectrum of Au/CeO_2_ differed from the spectrum of CeO_2_ nanorods mainly by the main peak at 450–460 cm^−1^ assigned to oxygen ions vibrational mode in the CeO_2_ structure (Figure S3). This peak was strongly broadened in Au/CeO_2_, which was caused by the smaller size of the crystalline domains due to the introduction of Au into the CeO_2_ structure. Besides, the Raman mode near 550 cm^−1^and 600 cm^−1^ assigned to the presence of oxygen vacancies was more intense for Au/CeO_2_ relative to the CeO_2_ nanorods, in agreement with the Au-CeO_2_ metal-support interactions that increased the number of oxygen vacancy sites (Figure S3) [26,27]. This is further confirmed by the Au 4f core-level XPS for Au/CeO_2_ nanorods (Figure S4). The Au 4f region showed two photoelectron peaks ascribed to Au 4f_7/2_ and Au 4f_5/2_ core-levels [12]. These peaks were shifted towards lower BE values, 82.7 and 86.3 eV, respectively, relative to Au in the metallic state [28]. The Ce 3d core-level XPS spectra showed peak positions characteristic of CeO_2_ with the presence of Ce^3+^ in both CeO_2_ and Au/CeO_2_ samples (Figure S5, Tables S1 and S2). The BET surface area for the samples CeO_2_ and Au/CeO_2_ were 86.3 m^2^·g^−1^ and 70.6 m^2^·g^−1^ (Figure S6 and Table S3), respectively. The comparable values evidence that the Au deposition did not affect the CeO_2_ nanorods surface area. The same trend was observed for the pore diameter distribution (Figure S7 and Table S3).

**Figure 2:**
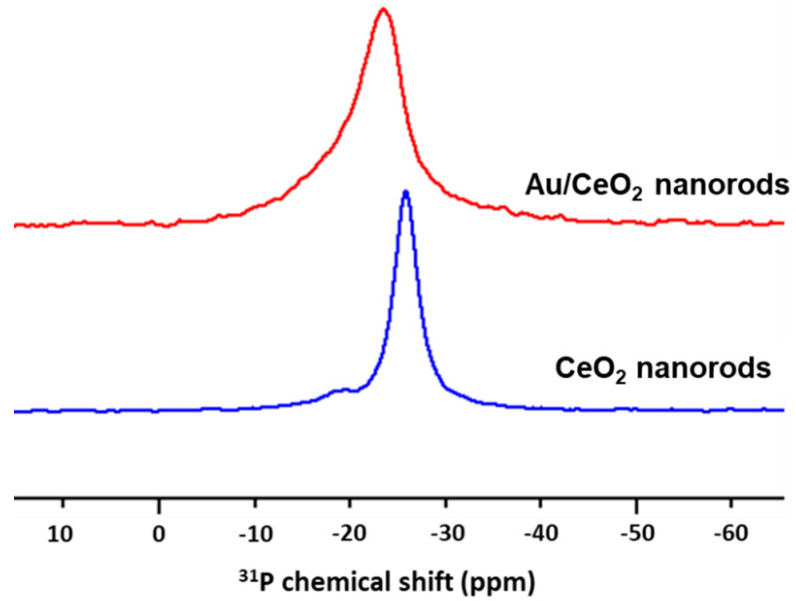
^31^P ss NMR spectra of CeO_2_ and Au/CeO_2_ nanorods (blue and red traces, respectively) using trimethylphosphine (TMP) as a surface probe. After Au deposition, the ^31^P NMR signal shifted from 25.7 to 23.5 ppm, suggesting that the electron density of the Ce surface is decreased upon Au deposition.

We also studied the Au/CeO_2_ and CeO_2_ nanorods by ^31^P-ssNMR analysis employing trimethylphosphine (TMP) as a surface probe. TMP is an electron donor molecule that can specifically form an adduct with surface Ce cation (i.e., TMP-Ce) [29]. In this case, the chemical shift of ^31^P is expected to differentiate the strength of the adduct bond formed with various Ce acidities (or electron density) as the stronger surface Lewis acid site pushes δ^31^P to positive ppm [30]. As shown in Figure 2, the ^31^P chemical shift of TMP-adsorbed nanorods shifted from 25.7 to 23.5 ppm after Au incorporation, suggesting that the electron density of surface Ce was decreased upon contact with Au. This lower electron density of surface Ce for the Au/CeO_2_ nanorods sample might be associated with more oxygen vacancies and more active vacancies [31,32].

We then turned our attention to the catalytic activities towards the tandem imine production from alcohols and amines driven by light irradiation. We employed only benzyl alcohol and aniline as substrates [21]. It is accepted that this tandem reaction pathway starts with the alcohol oxidation to aldehyde followed by a spontaneous aldehyde-amine coupling, releasing the active redox site for a new reaction [6]. We employed a white lamp as the irradiation source and started by investigating the activities of the individual Au NPs and CeO_2_ nanorods as catalysts as described in Table 1. The use of Au NPs as catalysts showed a very low conversion percentage under light excitation (2.3%, Entry 1), indicating that the LSPR excitation on Au NPs alone did not significantly contribute to the catalytic activity. The individual CeO_2_ nanorods (Table 1, Entries 2 and 3), on the other hand, displayed better activities, reaching around 62–63% conversion both in the dark and under light excitation. The higher activities occurred as a result of the presence of the oxygen vacancies, that acted as active sites for alcohol oxidation [8]. The similar activities detected for dark and light conditions show that the light excitation does not lead to any enhancements in catalytic activity in the CeO_2_ nanorods.

Interestingly, the incorporation of Au NPs into the CeO_2_ nanorods (Au/CeO_2_) led to a significant increase in the catalytic activity under light excitation to 91%, while keeping the selectivity to imines close to 97% (Table 1, Entry 4). In the dark, the activities corresponded to 69.5% (Table 1, Entry 5), which is close to the value detected for the individual CeO_2_ nanorods. The results from individual Au NPs (Entry 1) indicated that the enhancement observed for the Au/CeO_2_ sample under light irradiation (Entry 4) was not only due to the presence of Au NPs and its plasmonic effects, for example. The observed effect was necessarily due to the synergism between CeO_2_ nanorods and the Au NPs, which was responsible for a conversion increment far beyond the sum of the conversion promoted by the CeO_2_ nanorods and the Au NPs individual counterparts.

We performed control experiments using electron or hole scavengers (Table 1, Entries 8 and 9) and by replacing the reaction medium with an Ar atmosphere (absence of O_2_) (Table 1, Entries 6 and 7) to better understand the photocatalytic enhancements in Au/CeO_2_ nanorods. Under light excitation, the use of an Ar atmosphere decreased the conversion to 31.7% (Entry 6) relative to 91.2% in O_2_ (Entry 4). This demonstrates that the O_2_ plays a central role in this conversion driven by light. Suppression of catalytic activity was observed under the Ar atmosphere and light excitation relative to the utilization of the Ar atmosphere and dark conditions (62.1%, Entry 7). Surprisingly, under dark conditions, the conversion in the O_2_ and Ar atmosphere was similar (Entries 5 and 7, respectively). This result demonstrates that the presence of O_2_ and the plasmonic excitation (light irradiation) are required for the high activities detected for the Au/CeO_2_ nanorods (Entry 4).

**Figure 3:**
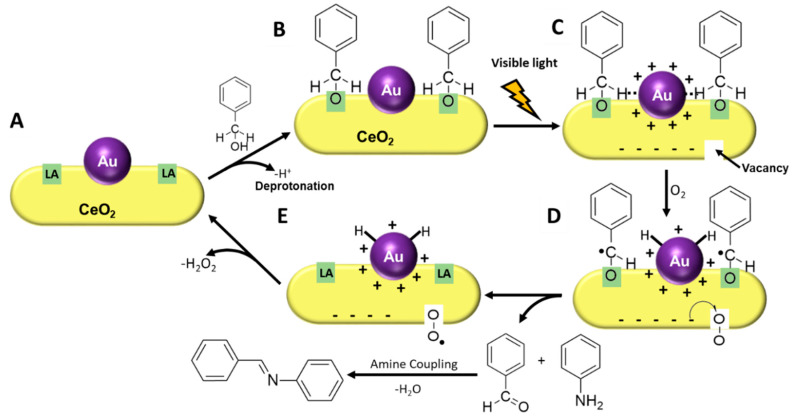
Schematic representation of the proposed photocatalytic mechanism. The LA sites (**A**) enable the adsorption of benzyl alcohol (**B**), via a deprotonation pathway. Localized surface plasmon resonance (LSPR) excitation leads to the formation of hot electrons and holes (**C**). Hot electrons get trapped at vacancy sites, where they activate adsorbed O_2_ (**C**,**D**). Hot holes facilitate the α-H abstraction from the adsorbed benzyl alcohol (**C**,**D**). The cerium-coordinated superoxide species can abstract hydrogen from H-Au species (**E**), regenerating the catalyst surface. The formed benzaldehyde couples with aniline in the next step to generate the imine.

Based on these results we would like to propose that the unique structure of the (110) surface facets in the CeO_2_ nanorods (Figure 3A) allow first the adsorption of benzyl alcohol (via a deprotonation pathway) (Figure 3B). As Tamura and collaborators demonstrated, this deprotonation step does not depend on O_2_ [6] and is easily accomplished by the Lewis acid (LA) sites on the CeO_2_ (110) surface facet (i.e., Ce^4+^ surficial species) [29,33,34]. Even under dark conditions some synergistic effect is observed for the Au/CeO_2_, the Au NPs promotes an increment from 62.5% (Entry 3) to 69.5% (Entry 5) for the imine formation. It is well-established for alcohol oxidation investigations (not light-driven) that this synergistic effect occurs due to the charge separation in the interface Au/CeO_2_, in which the Au NPs gives an e^−^ for the Ce^4+^ reducing it to Ce^3+^ and the Au NPs keep the positive charge [33,35]. These positive-charged Au species are responsible for the C-H activation, which is the limiting step of the reaction [33,35]. Under light irradiation, this synergistic effect, due to the charge separation, is further promoted. Upon LSPR excitation of the Au NPs, hot electrons and hot holes are generated [13,14]. The hot electrons can be trapped at the redox sites of the CeO_2_ while the hot holes remain at the Au NPs (Figure 3C and Figure S8). The electrons in the CeO_2_ will reduce some Ce^4+^ into Ce^3+^ generating vacancies (Figure 3C). In the next step, hot holes on Au NPs can contribute to the α-H abstraction from the adsorbed benzyl alcohol substrate leading to a carbon-centered radical (Figure 3C,D) and H-Au species over the Au NP [35,36,37]. These H-Au species were trapped by 5,5-dimethyl-1-pyrroline *N*-oxide (DMPO) in electron paramagnetic resonance (EPR) experiments reported by Garcia et al. under similar conditions [35]. Simultaneously, trapped hot electrons can be transferred to O_2_ species adsorbed on vacancies to give surface-bound •O_2_^−^ (Figure 3C,D). We then hypothesize that the carbon-centered radical can recombine with the electrons trapped on the vacancies (Ce^3+^ species) regenerating the Ce^4+^ and forming benzaldehyde, which couples with the aniline enabling the tandem formation of the imines. The cerium-coordinated superoxide species have the important role of regenerating the surface of Au NPs by hydrogen abstraction from H-Au (Figure 3E), leading to H_2_O_2_ [33]. By replacing O_2_ with an inert atmosphere, there is a decrease in the conversion percentage as no O_2_ is available to regenerate the Au surface. The deactivation of the Au surface in the absence of an oxidant can be noticed by comparing the reactions for the Au/nanorods sample under Ar (Entry 7) and nanorods (no Au) without irradiation under O_2_ (Entry 2), both display similar activities (62.1% and 62.9%, respectively), which are lower than the Au/nanorods sample under O_2_ (Entry 7). Garcia et al. reported that this reaction can be performed in the absence of O_2_ when DMPO is used to abstract the H-Au species and regenerate the Au NPs [35]. Therefore, it is plausible that in the absence of an oxidant, chemisorbed hydrogens stay bonded to the Au NPs surface, poisoning its surface. This is supported by the chromatogram for the reaction products in the absence of O_2_, which displayed the same profile and no amines were detected.

In the absence of O_2_ and under light the catalyst activity is suppressed, reaching smaller values relative to the reaction in the dark (both in the presence of O_2_ and Ar atmosphere). It is plausible that hot electrons trapped at the oxygen vacancy sites are not transferred to adsorbed O_2_. Instead, they can be transferred to the adsorbed benzyl alcohol species disfavoring the α-H abstraction and suppressing the reaction rates. Under dark conditions, the LSPR excitation does not play a role in this transformation and activities under Ar and O_2_ yield similar results. Thus, in the absence of light excitation, the O_2_ plays an even more important role over the benzyl alcohol oxidation. 

This proposed reaction pathway is also supported by the conversion percentage for the Au/CeO_2_ nanorods in the presence of electrons or hole scavengers (under light excitation and O_2_ atmosphere). In the presence of electron scavengers, adsorbed O_2_ species activation is disfavored and the resulting activities are similar for the Au/CeO_2_ nanorods under dark conditions and CeO_2_ nanorods. In this case, activities are dictated by the CeO_2_ redox sites. In the presence of hole scavengers, the α-H abstraction from the adsorbed benzyl alcohol substrate is suppressed, and conversion percentages decrease to 30.7%. Hole scavengers can deactivate the positive charged Au species as well as the CeO_2_ redox sites, both active sites present in the catalyst. This result suggests that the synergistic effect is taking place through a charge separation process and not only by a heating effect promoted by the Au NPs. The possibility of a reaction between EDTA (hole scavenger) and the reactants was excluded by the absence of new peaks in the GC-MS analysis. Under Ar atmosphere, activity suppression is also observed, this effect is related to the deactivation of the Au surface, once •O_2_^−^ formation is avoided and the catalyst surface is not regenerated. Furthermore, the absence of O_2_ or electron receptor species might influence the Ce^3+^/Ce^4+^ ratio, leading to a deactivation of the redox sites of the CeO_2_ [6].

## 4. Conclusions

In summary, we have demonstrated that the plasmonic excitation in Au/CeO_2_ nanorods enables an alternative reaction pathway that leads to improved catalytic performance relative to individual CeO_2_ nanorods towards the visible-light-driven tandem synthesis of imines via alcohol oxidation. Our results demonstrated that the presence of O_2_, oxygen vacancies in CeO_2_, and both hot electrons and holes generated as a result of LSPR excitation in the visible range were crucial to the detected photocatalytic performances. While the hot electrons lead to the formation of oxygen vacancies, enabling the adsorption of O_2_, hot holes generate positive-charged Au species, which facilitate the α-H abstraction from the adsorbed benzyl alcohol. The produced carbon-centered radical can recombine with the electrons trapped in the vacancies producing the benzaldehyde, which couples with the aniline, enabling the tandem formation of the imines. The cerium-coordinated superoxide regenerates the surface of Au NPs by hydrogen abstraction, forming H_2_O_2_. We believe the results presented herein can inspire the development of photocatalysts containing plasmonic components that can enable improved performance and selectivity via reaction pathways that are not accessible in semiconducting NPs or external heating.

## Figures and Tables

**Table 1:**
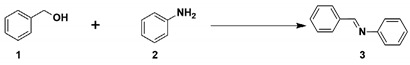
Conversion and selectivity for the tandem conversion of benzyl alcohol into imines as a function of the catalysts and reaction conditions.

Entry	Catalyst	Conversion of BnOH (%)	Selectivity (%)	
			Imine	Other
1 ^a^	Au NPs	2.3	100	0
	(Light)			
2	Nanorods	62.9	96	4
	(Light)			
3	Nanorods	62.5	97	3
	(No light)			
4	Au/nanorods	91.2	97	3
	(Light)			
5	Au/nanorods	69.5	97	3
	(No light)			
6 ^d^	Au/nanorods	31.7	97	3
	(Light, Ar atmosphere)			
7 ^d^	Au/nanorods	62.1	97	3
	(No light, Ar atmosphere)			
8 ^b^	Au/nanorods	68.7	97	3
	(Light, electron scavenger)			
9 ^c^	Au/nanorods	30.7	97	3
	(Light, hole scavenger)			

Typical experiment: 1 mmol benzyl alcohol, 2 mmol aniline, 50 mg of catalyst, 1 bar of O_2_, visible light, 50 °C, 48 h; ^a^ Au NPs colloidal suspension was added in equivalent amount to the Au concentration on Au/CeO_2_ samples; ^b^ 10 mmol benzoquinone (electron scavenger); ^c^ 10 mmol EDTA (hole scavenger); ^d^ Inert atmosphere (oxygen gas is replaced by argon).

## Supplementary Materials

The following are available online at https://www.mdpi.com/2079-4991/10/8/1530/s1, Figure S1: TEM CeO_2_ nanorods, Figure S2: XRD, Figure S3: Raman spectra, Figure S4 and S5: XPS spectra, Table S1: Binding energies Au 4f XPS, Table S2: Binding energies O 1s XPS, Figure S6: Isotherms BET, Figure S7: BJH pore diameter distribution, Table S3: BET surface area and average pore diameter, Figure S8: Scheme of the energy level diagram for the Au/CeO_2_ nanorod photocatalyst.
