# Supplementary material for: Hot Electrons, Hot Holes, or Both? Tandem Synthesis of Imines Driven by the Plasmonic Excitation in Au/CeO2 Nanorods
Source: Nanomaterials (Basel). 2020 Aug 4;10(8):1530. doi: 10.3390/nano10081530 (PMC7466498; doi:10.3390/nano10081530)
Supplement: Supplementary file 1 [file nanomaterials-10-01530-s001.pdf]

nanomaterials

Supplementary Materials

Hot Electrons, Hot Holes, or Both? Tandem Synthesis of Imines Driven by the Plasmonic Excitation in Au/CeO_2 Nanorods

Ivo F. Teixeira [1,*], Mauricio S. Homsi [2], Rafael S. Geonmonond [2], Guilherme F. S. R. Rocha [2], Yung-Kang Peng [3], Ingrid F. Silva [4], Jhon Quiroz [5] and Pedro H. C. Camargo [2,5,*]

[1] Department of Chemistry, Federal University of São Carlos, São Carlos 13565-905, Brazil
[2] Department of Fundamental Chemistry, Institute of Chemistry, University of São Paulo, São Paulo 05508-000, Brazil; mauriciosamuel.homsi@usp.br (M.S.H.); rafaelsg31@gmail.com (R.S.G.); guilhermefrrocha@gmail.com (G.F.S.R.R.)
[3] Department of Chemistry, City University of Hong Kong, Yeung Kin Man Academic Building, Hong Kong, China; ykpeng@cityu.edu.hk
[4] Department of Chemistry, ICEx, Universidade Federal de Minas Gerais, Belo Horizonte 31270-901, Brazil; ingridfs@ufmg.br
[5] Department of Chemistry, University of Helsinki, A.I. Virtasen aukio 1, 00100 Helsinki, Finland; jhon.quiroz@helsinki.fi
* Correspondence: ivo@ufscar.br (I.F.T.); pedro.camargo@helsinki.fi (P.H.C.C.)

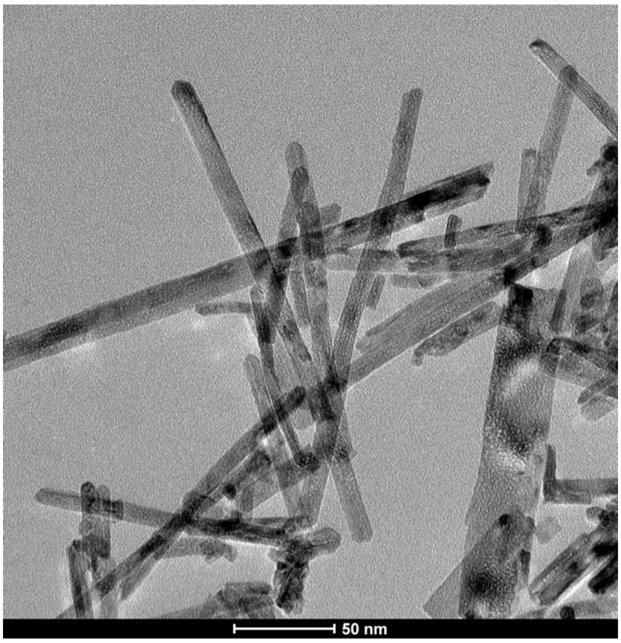

Figure S1. TEM images for the CeO_2 nanorods employed as starting materials for the synthesis of Au/CeO_2 nanorods.

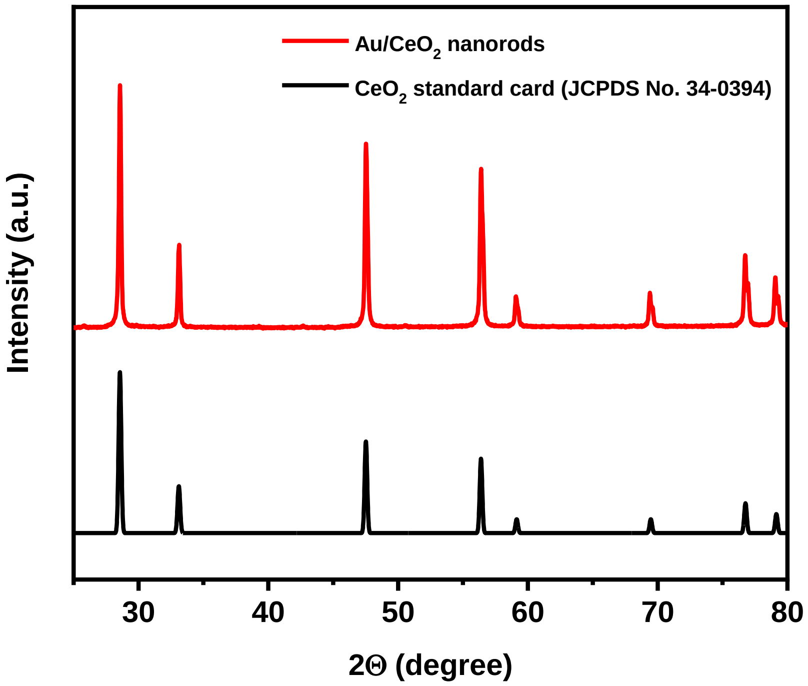

Figure S2. XRD pattern for Au/CeO_2 nanorods. The X-ray diffractometry evidenced the good crystallinity of the sample with peaks assigned to CeO_2 phase.

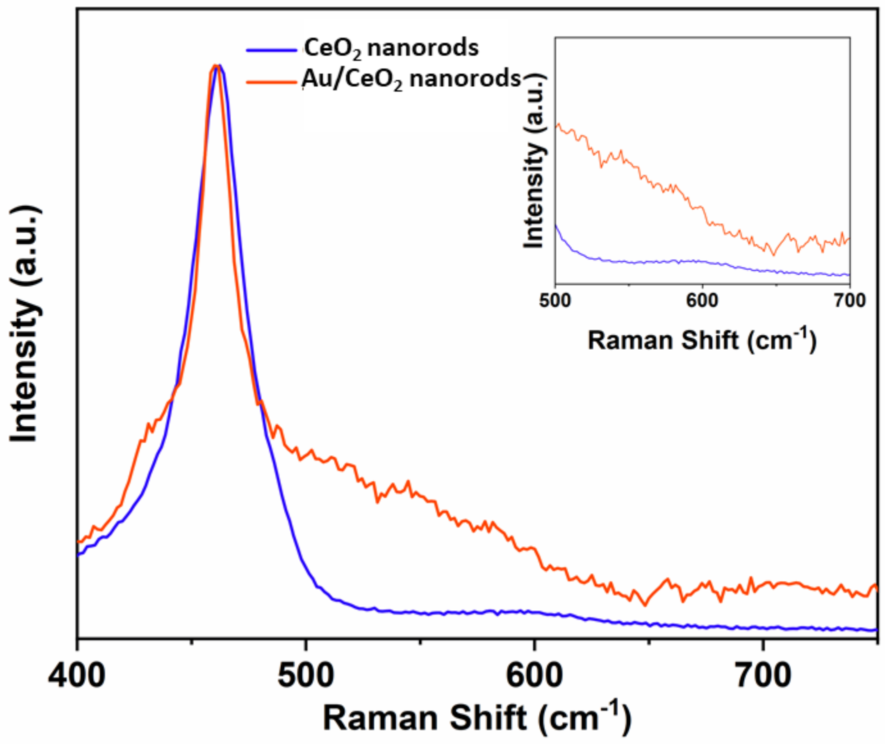

Figure S3. Raman spectra for CeO_2 (blue trace) and Au/CeO_2 (red trace) nanorods. The Raman spectrum of Au/CeO_2 displays a shoulder at 550–600 cm^{-1} assigned to the presence of oxygen vacancies, which is more intense for the Au/CeO_2 nanomaterials relative to the CeO_2 nanorods.

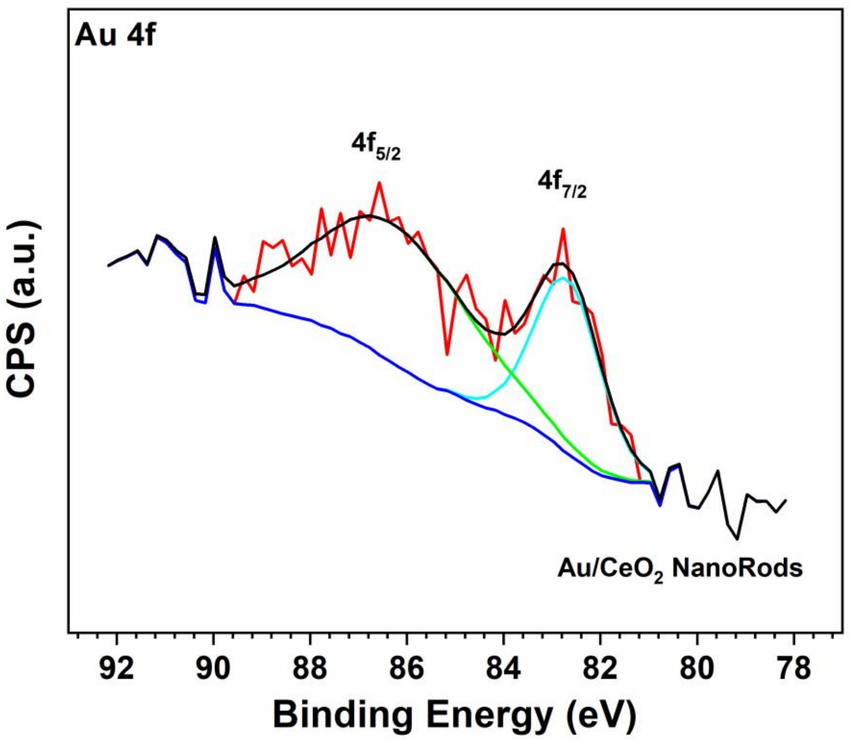

Figure S4. XPS spectra of the Au 4f core-levels for Au/CeO_2 Au/CeO_2 nanorods. It can be observed that for the nanorods, the Au signal is shifted to higher binding energies as a result of stronger metal-support interaction.

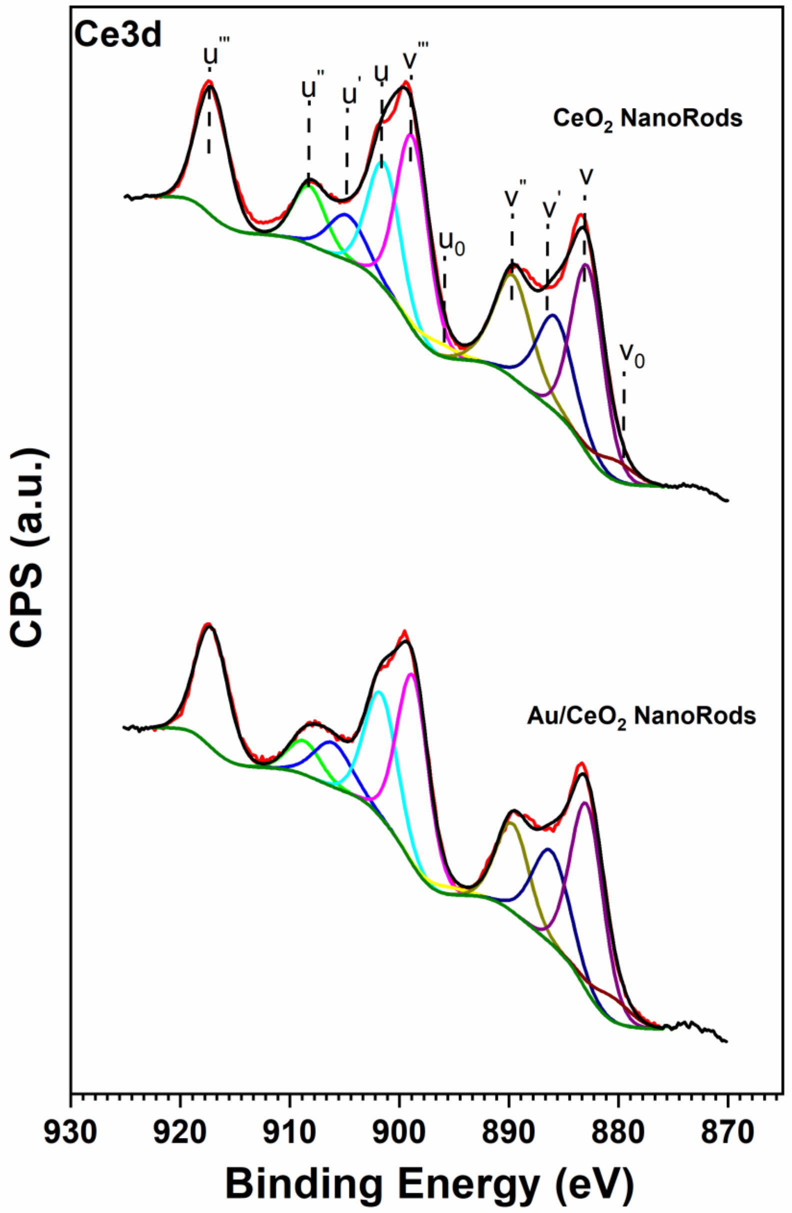

Figure S5. XPS spectra of the Ce 3d core levels for CeO_2 nanorods and Au/CeO_2 nanorods (top to bottom, respectively). No significant differences in the signals for Ce^{3+} and Ce^{4+} species were detected in the samples. They can be associated with different profiles: Ce^{3+} (v_0, v′, u_0 and u′) and Ce^{4+} (v, v″, v‴, u, u″, and u‴). The estimated percentages of Ce^{3+} and Ce^{4+} ions reveal a minimal variance among the samples, suggesting that the addition of low quantities of gold is not promoting the presence of Ce^{3+} and consequently the creation of oxygen vacancies on the cerium oxide as a result of the metal-support interaction [7].

Table S1. Binding energies (eV) of peak positions from Au 4f region and the atomic ratio of Au/Ce for the Au/CeO_2 nanorods.

**Sample**	**$4f_{7/2}$**	**$4f_{5/2}$**	**Au/Ce**	**%at Ce**	**%at Au**
Au/CeO_2 nanorods	82.7	86.3	0.009	99.1	0.9

Table S2. Binding Energy (eV) of O 1s components of samples and relative atomic ratio among species (at. %). Table S2 depicts the BE values obtained from the XPS spectra for the O 1s region for pure CeO_2 and Au/CeO_2 samples, respectively. The signal was deconvoluted into three components located at around 529, 531 and 533 eV (Table S2). These are assigned to lattice oxygen (O_L), oxygen vacancies or surface oxygen ions (O_S), and adsorbed water (O_W), respectively [8]. For all samples, the signal from O_S had the highest contribution with 58% for individual CeO_2 nanorods. After the addition of Au, a slight decrease of the O_S contribution was registered. These results indicate that the enrichment of oxygen vacancies or oxygen ions on the surface of CeO_2 is strongly associated with the shape of the oxide rather than by the addition of Au. It is noteworthy that the Au loading in these samples is very low.

**Sample**	**O 1s B.E. (at. %)**		
	**O_L**	**O_S**	**O_W**
Au/CeO_2 nanorods	529.1 (28)	530.8 (45)	533.2 (27)
CeO_2 Nanorods	528.5 (23)	530.4 (58)	532.7 (19)

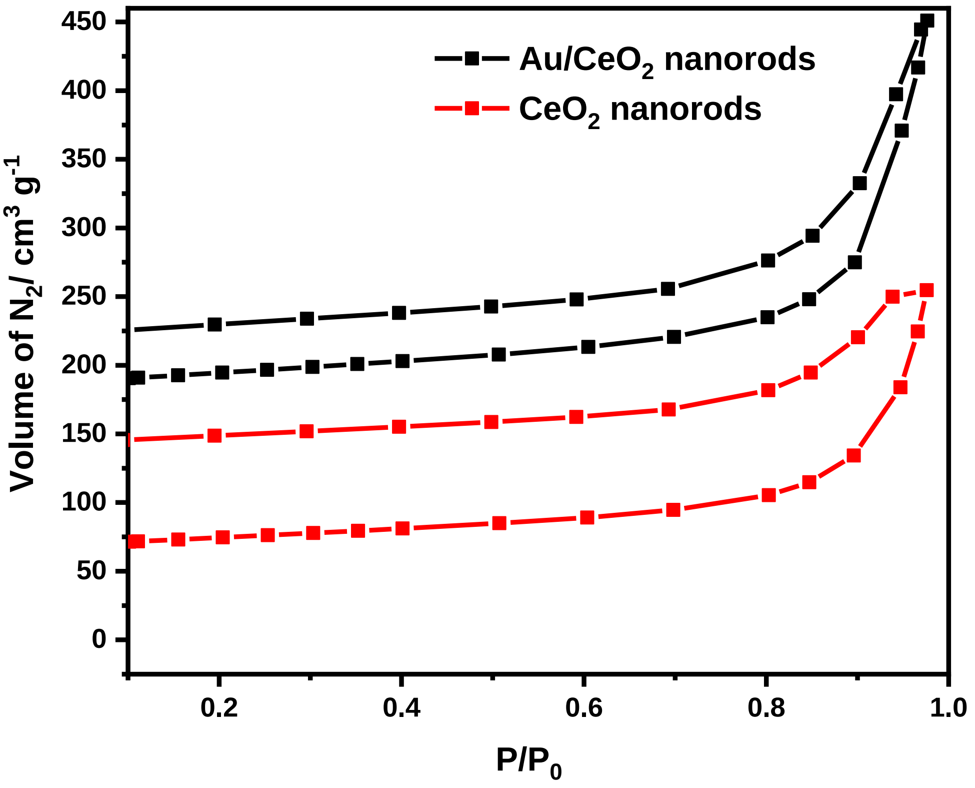

Figure S6. Isotherms BET for CeO_2 nanorods (red trace) and Au/CeO_2 nanorods (black trace). The CeO_2 nanorods samples display a similar porosity and surface area when compared with the Au/CeO_2 nanorods.

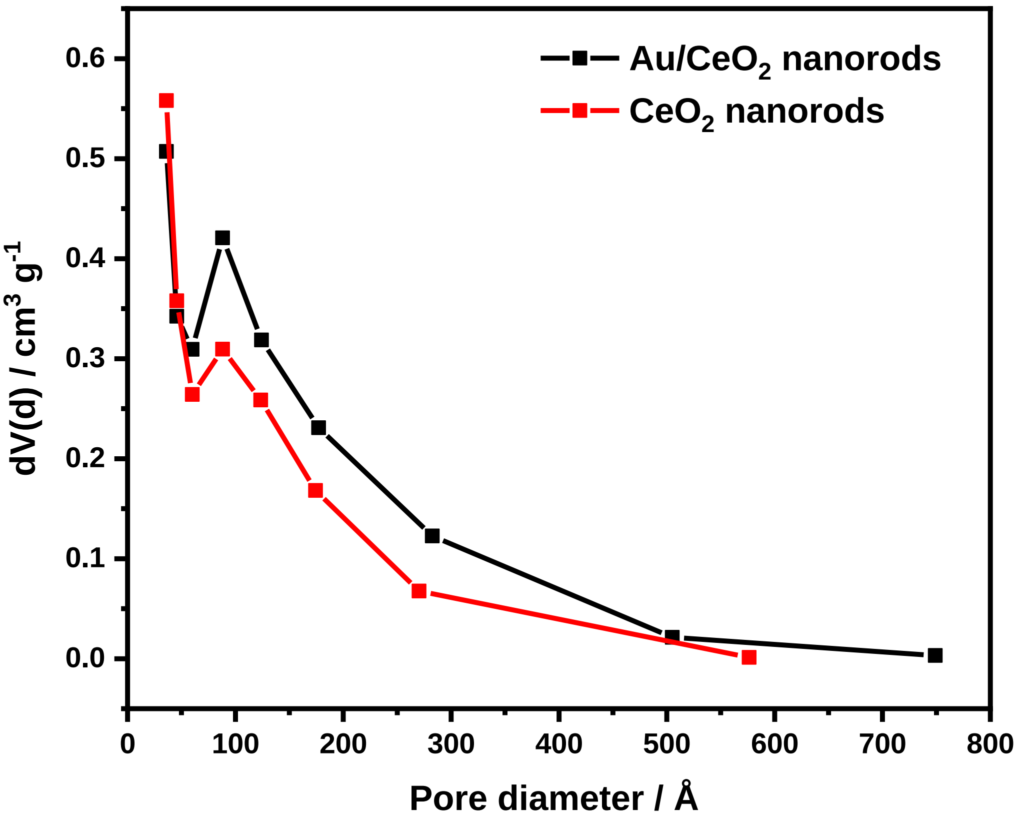

Figure S7. BJH pore diameter distribution for the for CeO_2 nanorods (red trace) and Au/CeO_2 nanorods (black trace). The CeO_2 nanorods samples present mainly mesoporous with larger pores diameters.

Table S3. BET surface area and average pore diameter from Figures S6 and S7.

Sample	Area BET ($m^2 \cdot g^{-1}$)	Pore Diameter (Å)
Au/CeO_2 Nanorods	86.3	177.2
CeO_2 Nanorods	70.6	123.7

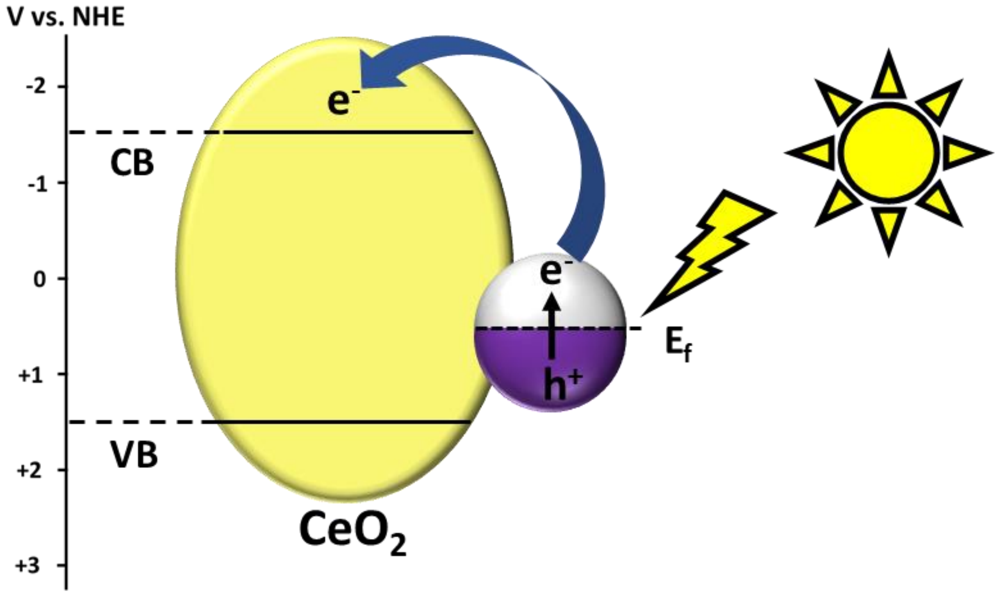

Figure S8. Energy level diagram for the Au/CeO_2 nanorod photocatalyst, illustrating the injection of hot electrons on the conduction band of the CeO_2 and the generation of hot roles in the Au NPs.

© 2020 by the authors. Submitted for possible open access publication under the terms and conditions of the Creative Commons Attribution (CC BY) license (http://creativecommons.org/licenses/by/4.0/).
